# Protein Adsorption, Calcium-Binding Ability, and Biocompatibility of Silver Nanoparticle-Loaded Polyvinyl Alcohol (PVA) Hydrogels Using Bone Marrow-Derived Mesenchymal Stem Cells

**DOI:** 10.3390/pharmaceutics15071843

**Published:** 2023-06-28

**Authors:** Jeevithan Elango, Camilo Zamora-Ledezma, Frank Alexis, Wenhui Wu, José Eduardo Maté-Sánchez de Val

**Affiliations:** 1 Department of Biomaterials Engineering, Faculty of Health Sciences, UCAM—Universidad Católica San Antonio de Murcia, Campus de los Jerónimos 135, Guadalupe, 30107 Murcia, Spain; srijeevithan@gmail.com; 2 Center of Molecular Medicine and Diagnostics (COMManD), Department of Biochemistry, Saveetha Dental College and Hospitals, Saveetha Institute of Medical and Technical Sciences, Saveetha University, Chennai 600077, India; 3 Department of Marine Bio-Pharmacology, College of Food Science and Technology, Shanghai Ocean University, Shanghai 201306, China; 4 Green and Innovative Technologies for Food, Environment and Bioengineering Research Group (FEnBeT), Faculty of Pharmacy and Nutrition, UCAM—Universidad Católica San Antonio de Murcia, Campus de los Jerónimos 135, Guadalupe, 30107 Murcia, Spain; czamora9@ucam.edu; 5 Departmento de Ingenería Química, Colegio de Ciencias y Ingenierias, Universidad San Francisco de Quito (Ecuador), Campus Cumbayá, Diego de Robles s/n, Quito 170901, Ecuador; falexis@usfq.edu.ec

**Keywords:** hydrogel, PVA, AgNPs, mesenchymal stem cells, protein and mineral interaction

## Abstract

Several approaches have evolved to facilitate the exploration of hydrogel systems in biomedical research. In this sense, poly(vinyl alcohol) (PVA) has been widely used in hydrogel (HG) fabrication for several therapeutic applications. The biological properties of PVA hydrogels (PVA-HGs) are highly dependent on their interaction with protein receptors and extracellular matrix (mainly calcium) deposition, for which there is not enough evidence from existing research yet. Thus, for the first time, the functional properties, like protein and mineral interactions, related to the proliferation of mesenchymal stem cells (MSCs) by silver nanoparticle (AgNP)-loaded PVA hydrogels (AgNPs-PVA-HGs) were investigated in the present study. The UV absorption spectrum and TEM microscopic results showed a maximum absorbance of synthesized AgNPs at 409 nm, with an average particle size of 14.5 ± 2.5 nm, respectively. The functional properties, such as the calcium-binding and the protein adsorption of PVA-HG, were accelerated by incorporating AgNPs; however, the swelling properties of the HGs were reduced by AgNPs, which might be due to the masking of the free functional groups (hydroxyl groups of PVA) by AgNPs. SEM images showed the presence of AgNPs with a more porous structure in the HGs. The proliferative effect of MSCs increased over culture time from day 1 to day 7, and the cell proliferative effect was upregulated by HGs with more pronounced AgNPs-PVA-HG. In addition, both HGs did not produce any significant cytotoxicity in the MSCs. The histological (bright light and H&E staining) and fluorescence microscopic images showed the presence of a cytoskeleton and the fibrillar structure of the MSCs, and the cells adhered more firmly to all HGs. More fibrillar bipolar and dense fibrillar structures were seen in the day 1 and day 7 cultures, respectively. Interestingly, the MSCs cultured on AgNPs-PVA-HG produced extracellular matrix deposition on day 7. Accordingly, the present results proved the biocompatibility of AgNPs-PVA-HG as a suitable system for culturing mammalian stem cells for regenerative tissue applications.

## 1. Introduction

Hydrogels (HGs) are formed by the hydrophilic interaction of water and polymers, which produce a three-dimensional network that facilitates the swelling ratio in a significant amount upon interaction with water [1,2,3]. The polymers used for hydrogel fabrication should be hydrophilic, easy to crosslink, tend to swell, and be low in toxicity without side effects. Due to their wettability, the hydrogels have been potentially used in several therapeutic applications, such as wound healing and the regeneration of skin, bone, dental and cartilage, as well as drug delivery and cancer treatment [4,5,6]. Mechanically stronger and tough hydrogels were fabricated to compensate for the load-bearing ability of cartilage replacement, artificial muscles, artificial organs, and contact lenses [7]. Several polymers, such as collagen [8,9], chitosan [10,11,12], alginate [13,14], poly(Vinyl Alcohol) (PVA) [15], polyLactic Acid (PLA) [16], silk fibroin [17], and cellulose [18] has been widely used in hydrogel fabrication for regenerative tissue purposes.

Among the different polymers, naturally derived biopolymer-based hydrogels had poor mechanical and chemical stability, which limits their potential application in tissue regeneration. In this sense, the use of PVA in hydrogel fabrication overcomes the limitation of natural hydrogels in practical application. As a hydrophilic polymer, PVA is considered a suitable biocompatible synthetic material, which has the major functional hydroxyl groups. The presence of a hydroxyl group makes PVA more readily available for crosslinking with several polymers to form hydrogels [19]. Due to their unique structure and mechanical properties, PVA hydrogels are considered excellent candidates and have great potential applications in many fields, including but not limited to tissue engineering [15].

Numerous studies investigated the fabrication of composite PVA hydrogels associated with different natural biopolymers, such as alginate, cellulose, ovalbumin, dextran, heparin, gelatin, collagen, hyaluronic acid, chondroitin sulfate, chitosan, silk fibroin, starch, fibrin, gellan, and carrageenan and synthetic polymers, such as ultra-high molecular weight poly-ethylene (UHMWPE), poly (acrylic acid), polydimethyl siloxane, polyurethanes, poly(N-isopropyl acrylamide), poly (vinyl pyrrolidone), poly (glycolic acid), poly (D, L-lactide-co-glycolide), bioceramics (calcium phosphates, magnesium phosphate, tricalcium phosphate, substituted apatite, hydroxyapatite, and biphasic systems), and their potential in tissue engineering applications [20].

The use of silver nanoparticles (AgNPs) is of great interest in the field of nanotechnology due to its optical, electrical, catalytic, and biofunctionality properties. In particular, both their physicochemical properties and biological functionality would dramatically depend on their morphology, size, and distribution, as well as on their electrochemical environment in the case of nanoparticles in a liquid medium. Indeed, AgNPs belong to the most commonly used nanomaterials in biomedical applications mainly due to their antimicrobial potential, but they are also very promising against nonliving organisms, including human immunodeficiency viruses that can induce several viral diseases [21]. In this way, it is particularly important to manufacture AgNPs in a controlled manner for the development of biomedical applications such as detection devices, drug delivery systems, and wound dressings, among others. Additionally, for the most important parameters that influence the morphology of the AgNPs during their synthesis, the following stand out: (i) the choice of reducing agent, (ii) relative amounts (iii) reagent concentrations, and (iv) the temperature and duration of the reaction [22,23]. Although PVA and AgNPs have been widely used for hydrogel fabrication and tested for several applications, most are focused on the development of antibacterial/antifouling biomedical materials. However, the major scientific gap between PVA hydrogel and its therapeutic application is understanding the ability of PVA hydrogels to interact with cellular plasma protein and extracellular matrix components. Mainly, calcium should be addressed for a better understanding of the fundamental concept of PVA and AgNPs in practical applications. Unfortunately, none of the studies explored the possible interaction mechanism of PVA and AgNP hydrogels in biological cells. Considering the above hypothesis, the present study fabricated AgNP-loaded PVA hydrogel (AgNPs-PVA-HG) and investigated the influence of AgNPs in PVA hydrogel interactions with plasma proteins and their calcium binding ability in relation to the proliferation ability of AgNPs-PVA-HG in bone marrow mesenchymal stem cells.

## 2. Materials and Methods

### 2.1. Materials

Polyvinyl alcohol (PVA) molecular weight 9000–10,000 g/mol (CAS: 9002-89-5), AgNO_3_ (silver nitrate, CAS: 7761-88-8) molecular weight 169.87 g/mol, NaBH_4_ (sodium borohydride, CAS: 16940-66-2) molecular weight 37.83 g/mol and were acquired from Sigma-Aldrich (St. Louis, MO, USA). Liquid vegetable glycerin, pharmaceutical grade, was acquired from EQM chemical solution store (Madrid, Spain).

### 2.2. Fabrication of PVA-AgNP Hydrogels

Hydrogels were fabricated as follows. First of all, AgNPs were synthesized by the chemical reduction method [23,24,25], using AgNO_3_ (silver nitrate) as the initial reagent and NaBH_4_ (sodium borohydride) as the reducing agent. The corresponding chemical reaction.

Ag[NO]_3_ + NaBH_4_ → Ag + H + BH_3_ + NaNO_3_

During the AgNPs synthesis process, the NaBH_4_ adsorption plays a crucial dual role as a reducing agent but also as a stabilizer during the growth of silver (Ag) nanoparticles, providing a surface charge on the particle. Thus, nanoparticles are created and, at the same time, stabilized in suspension through repulsive electrostatic forces between them due to adsorbed NaBH_4_. Besides, the amount of NaBH_4_ must be controlled. Indeed, it must be enough to stabilize the particles when the reaction occurs, but not too high; otherwise, the ionic strength will increase, promoting the aggregation of the particles. The stability of these solutions strongly depends on the proportion of reducing agents and varies between minutes and/or weeks. In fact, this route offers the fabrication of NPs with diameters ranging from ~10 nm to a few dozen of nm, with a Surface Plasmon Resonance (SPR) around ~400 nm and full width at half maximum around ~35–100 nm. If the amount of reductant is not adequate, the particles agglomerate, and the solution turns yellow, violet, to gray. The latter aggregation can be avoided by adding dispersing/stabilizing agents such as polyvinylpyrrolidone (PVP).

**Figure 1:**
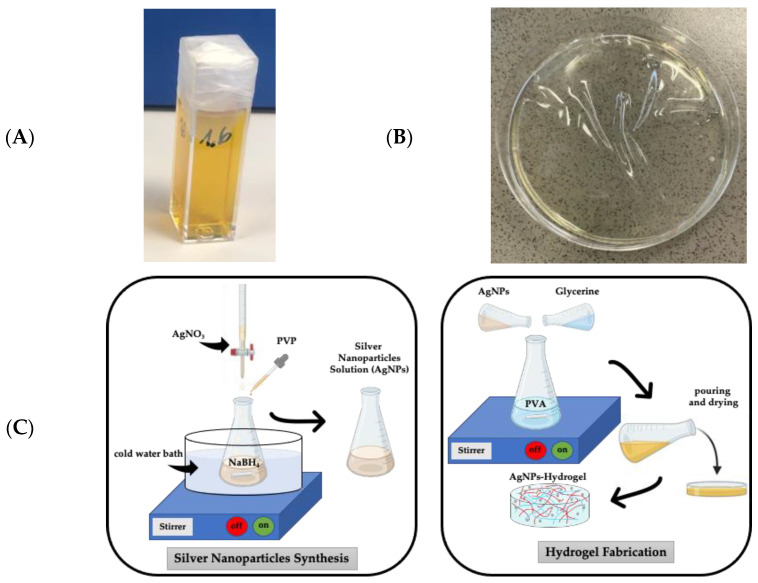
(**A**) Typical vial containing 4 mL of silver nanoparticles in solution. (**B**) Typical 120 mm Petri dish containing an AgNPs hydrogel (H-AgNPs). For clarity, the hydrogel has been intentionally wrinkled. (**C**) Schematic diagram of fabrication of hydrogels loaded with AgNPs.

For the synthesis of spherical AgNPs, 1.0 mM AgNO_3_ and 2.0 mM NaBH_4_ were prepared in distilled water beforehand. Typically, 10 mL of the AgNO_3_ solution is added dropwise (~1 drop/s or using a burette instead) to a vial containing 16 mL of the NaBH_4_ solution; in this way, the ratio [NaBH_4_]/[AgNO_3_] was 1.6. The whole reaction was carried out in an ice bath with vigorous stirring. The solution turns to yellow color at the end of the AgNO_3_ addition, and the whole process takes about ~3 to 5 min after which stirring is turned off. Just before switching off the agitation, 1 mL of an aqueous solution of PVP at 0.3 wt.% is also added drop by drop (see Figure 1C, in which a schematic diagram for the AgNPs fabrication is shown). Thus, the colloidal dispersion remains stable at room temperature for weeks to months. Figure 1 shows a typical macroscopic image of a vial containing 3 mL of AgNPs in solution.

In parallel, for the hydrogel, a PVA solution at 20 wt.% was prepared beforehand by pouring 20 g of PVA with 80 g of deionized water into a vial, followed by moderate stirring with a magnetic stirring plate for 24 h at 90 °C until a homogenous and transparent mixture was obtained. Finally, the AgNP hydrogel was fabricated by mixing (at room temperature for 15 min) the former PVA solution (8 g) with the silver nanoparticle solution (8 g) and the crosslinker, vegetal glycerin (4 g) in a ratio of 40/40/20 (Figure 1C, in which a schematic diagram for the hydrogel fabrication is shown). Then, the homogeneous mixture was poured into a Petri dish (120 mm of internal diameter) and left to dry at 40 °C for 72 h until the water evaporated. As a control sample, a hydrogel without AgNPs was fabricated by adding water instead of a silver nanoparticle solution. In Figure 1B, a representative macroscopic image of the AgNP hydrogel (H-AgNPs) peeled off from the substrate can be observed. For clarity, the hydrogel has been intentionally wrinkled.

### 2.3. Silver Nanoparticles Characterization

In order to characterize the silver nanoparticle’s size and morphology, we have performed UV-vis spectroscopy and transmission electron microscopy (TEM). All the UV-vis spectra recorded within the wavelength range of 300 nm to 700 nm and systematically background-corrected were recorded by using a UV-1800 spectrophotometer (Shimadzu Corporation, Tokyo, Japan) and using quartz cells with an optical path of d ≈ 10 mm. Spectra analysis was performed by using Peakfit v4.12 software. For TEM measurements, 20 µL of the AgNPs solution was deposited on a formvar/carbon-coated nickel-300 mesh grid. The TEM micrographs were obtained with an accelerating voltage of 120 kV by using a JEOL JEM 1011 (JEOL Ltd., Tokyo, Japan) Transmission Electron Microscope equipped with an Orius SC200 High Contrast Digital Camera (Gatan, Evry, France). For statistical analysis of the AgNPs size distribution, 100 measurements were performed using the ImageJ software. Subsequently, the silver nanoparticle’s size distribution was obtained by performing a Gaussian analysis of the histogram plotted using GraphPad Prism 9.0 Software, LLC.

### 2.4. Morphology of Silver Nanoparticles

Morphological characterizations of the PVA and PVA-AgNPs hydrogel were carried out by scanning electron microscopy (SEM; model JEOL-6100 (JEOL Ltd., Tokyo, Japan)), and energy dispersive X-ray spectroscopy (EDX; Oxford INCA (Oxford Instruments plc., Abington, Oxfordshire, UK)). For SEM/EDX analysis, the samples were freeze-dried and then deposited on aluminum sample holders with double-sided carbon tape. In order to guarantee the adequate conductivity of the sample, one drop of carbon ink was also used to fix the sample on the carbon tape. Then, the samples were coated with a platinum ~5 nm coating thickness conductive layer (99.99% purity) using a sputtering evaporator (BIORAD-POLARON). Sample images were collected at a variable magnification from 300× to 2000× with an accelerating voltage of 5 kV.

### 2.5. Water Holding Capacity

The water-holding property measured the ability of hydrogels to hold/retain water within the intramolecular spaces and was investigated by following our previous protocol [17,26]. Briefly, the hydrogel was weighed initially (Wi) and wetted with 1 mL distilled water for 3 h. Then the excess water from the hydrogel was gently removed and weighed again (Ww). The percentage of water holding capacity was calculated by using the below formula:

Water holding capacity (%) = (Ww − Wi)/Wi × 100

where Ww—wet weight of the hydrogel; Wi—initial weight of the hydrogel.

### 2.6. Swelling Ratio

The swelling measures the ability of hydrogels to expand their shape and size in an aqueous medium, and the percentage of the swelling ratio of hydrogel was calculated by following our previous method [26]. Briefly, the area of hydrogel was measured before swelling and noted as Ai, and then the hydrogel was incubated in distilled water for 24 h. Then the area of hydrogel was measured and noted as As. The percentage of swelling ratio was calculated by using the below formula:

Swelling ratio (%) = As − Ai/Ai × 100

where Ai—area of the initial hydrogel, and As—area of the swollen hydrogel.

### 2.7. Calcium-Binding Ability

The ability of hydrogel in mineral binding was evaluated by following our earlier methods [15,27]. Briefly, the hydrogel was incubated in calcium chloride (1 mg/mL) for 24 h, and the unbound calcium was washed out by phosphate-buffered saline (PBS). Then, the bound calcium on hydrogel was stained by using alizarin red-S, and the images were captured by a digital camera. The amount of calcium in the hydrogel was quantified by using an eluting solution (20% methanol and 10% acetic acid in water) at 450 nm using SpectraMax iD3 (Molecular Devices, LLC., San Jose, CA, USA) as per our earlier methods. For negative control, the hydrogels without calcium were treated in the same way as described. For blank control, the hydrogels without calcium coating were also stained with alizarin red-S dye.

### 2.8. Protein-Binding Ability

The ability of hydrogel in protein binding was done as per our previous protocols [27]. Briefly, the hydrogel was incubated in 200 µL fetal bovine serum (FBS) (Lot No. 2445724RP, Gibco, Carlsbad, CA, USA) for 2 h at 37 °C. Then, the unbound FBS was gently removed by washing with PBS twice and the gels were stained for bound protein on hydrogel using Coomassie Brilliant Blue G-250 (CBB G-250) dye for 4 h. After staining, the gels were again washed with de-staining solution (methanol/water/acetic acid) (Roche, Barcelone, Spain) for 30 min, twice. The stained gel images were taken by using a digital camera, and the amount of protein stain was quantified spectrometrically at 590 nm using SpectraMax iD3. For the blank control, the hydrogels without FBS treatment were also stained with CBB G-250. The cells without hydrogels were considered as control and treated in the same way as described.

### 2.9. Cell Culture

In vitro cell culture work was done by using Human Mesenchymal Stem Cells from Bone Marrow (MSCs). The cells were procured in the cryopreserved form from PromoCell GmbH, Heidelberg, Germany (Catalog Number C-12974, hMSC-BM-c, lot #1× 475Z011.3, Order No. AT240719). The cells were cultured as per the standard protocol using mesenchymal culture medium (PCS-500-030) with growth kit (PCS-500-041), 5% FBS (Gibco), 1% Penicillin-Streptomycin-Amphotericin B Solution (ATCC PCS-999-002). Initially, the cells were seeded in two T75 cm^2^ culture flasks and subcultured eight times on confluence. In each culture, some cells were stored for future use, and cells from subcultures 4 to 7 were used for the present study [28].

### 2.10. Cell Proliferation

For proliferation, the cells were seeded on hydrogel with a cell density of 1 × 10^4^ in 24 well culture plates. The cells cultured without hydrogel in the culture plate were considered as control. Both control cells and hydrogel groups were cultured with complete MSC culture medium in a CO_2_ incubator at 37 °C, 98% relative humidity, at 5% CO_2_ for 1, 3, and 7 days. On each period, the culture medium was removed, and the cells were treated with Alamar blue (Thermo Fisher Scientific (Waltham, MA, USA), Cat No: DAL1025) reagent (1:9 *v*/*v*) prepared in culture medium for 4 h at 37 °C. Then the cell viability was measured spectrophotometrically at 570 nm using SpectraMax iD3.

### 2.11. Cytotoxicity

For cytotoxicity, the cells were seeded the same as above in Section 2.9, and the control groups were the cells without hydrogels. All the treatments were carried out in triplicates. The cells were cultured for 1, 3, and 7 days with MSC culture medium in a CO_2_ incubator at 37 °C, 98% relative humidity, at 5% CO_2_. Then, the cells were washed with PBS to remove the existing culture medium, and a fresh culture medium containing 0.5 mg/mL 3-(4,5-dimethylthiazol-2-yl)-2,5-diphenyl tetrazolium bromide (Labclinics, Barcelona, Spain). The cells were incubated for 4 h in a CO_2_ incubator, the unbound yellow dye was removed gently by PBS wash, and the formed formazan dye was solubilized with DMSO, followed by measuring the OD at 570 nm using SpectraMax iD3.

### 2.12. Histological Staining

The morphology of the MSCs on hydrogel was visualized by using a histological staining method. In brief, the cells were cultured on hydrogel with a cell density of 5 × 10^4^ in 12-well culture plates using complete MSC culture medium for 1, 3, and 7 days. Each time, the cells were washed with PBS and fixed with 4% paraformaldehyde for 1 h at room temperature, followed by consecutive fixing with 2.5% glutaraldehyde for another 1 h at room temperature. The cells on the hydrogel were captured in white light using a fluorescence microscope coupled with Axiocam 305 mono (Axio Vert A1, Serial No 3847016567, Carl Zeiss Microscopy GmbH, Suzhou, China) before histological staining. Then, the cells on hydrogels were stained with hematoxylin stain for 30 min, incubated in bluing reagent, followed by eosin stain for 30 min. The bright field light images and HE-stained images were captured using an Axio Vert A1 microscope.

### 2.13. Fluorescence Microscopy

We further investigated the morphological structure of the MSCs cultured on the hydrogel via a fluorescence microscope. Briefly, the cells were seeded on hydrogels and cultured in a complete MSCs culture medium for 1, 3, and 7 days, as described in Section 2.11. After each treatment, the cells were fixed with 4% paraformaldehyde and 2.5% glutaraldehyde for 1 h each, respectively. The cell membrane was permeabilized with 0.1% Triton X-100 in PBS. Then, the cytoskeletal and cell nuclei were stained with fluorophores FITC and DAPI, following standard protocol. The images were captured at different magnifications by using an Axio Vert A1 fluorescence microscope. The cells cultured without hydrogels served as control.

### 2.14. Statistical Analysis

In the present study, all the experiments were carried out in three independent set-ups and the results were summarized as the mean and standard deviation. The statistical analysis and graph diagram were carried out by using Prism GraphPad 9.5.1. The statistically significant differences between groups were analyzed using a *t*-test, and a *p*-value less than 0.05 was considered statistically significant.

## 3. Results

The flexible and elastic properties of fabricated hydrogels are shown in Supplementary Video S1. In addition, the fabricated hydrogels were transparent and not opaque, which allowed the hydrogel to be a more efficient read-out platform for experimental purposes. The mechanical properties, such as elasticity, yield strength, young modulus, and concomitant physicochemical properties, can be readily modulated by adjusting the quantity of PVA and glycerine.

### 3.1. Silver Nanoparticles Characterization

**Figure 2:**
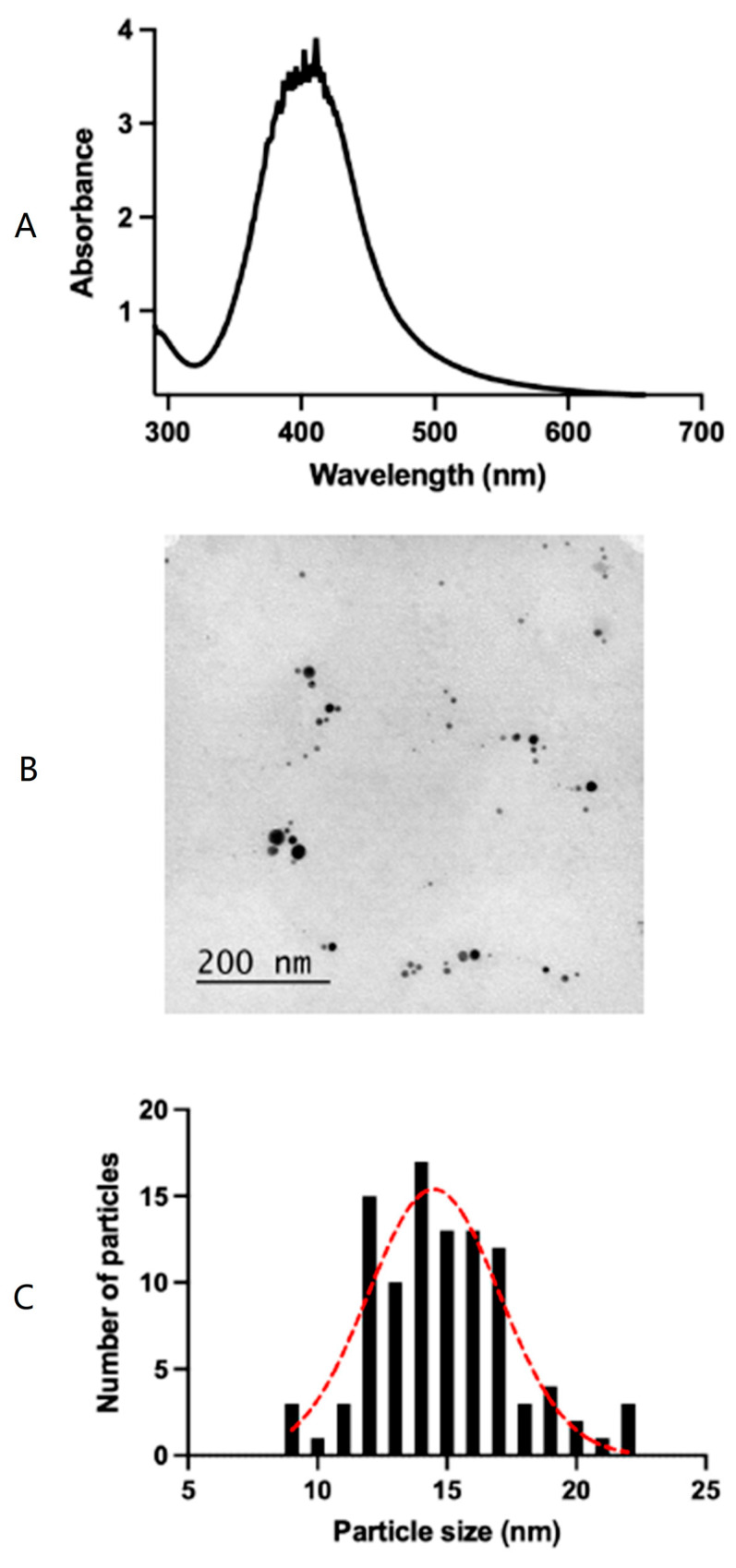
(**A**) Typical UV absorbance spectrum for AgNPs in solution with the maximum located at 409 nm. (**B**) Representative TEM image showing AgNPs. (**C**) Histogram of the silver nanoparticle size distribution (dotted red line corresponds to a Gaussian fit. Average AgNP size of 14.5 ± 2.5 nm).

The AgNPs’ spectrum absorbance can be used to indicate the average particle size. Indeed, the wavelength of the absorption maximum and its peak width at half maximum (PWHM) in each solvent for AgNPs provide useful information. Figure 2A shows a typical absorbance spectrum for the AgNPs synthesized with a maximum absorbance located at 409 nm with a peak width at half maximum (PWHM) of 81 nm, obtained from the peak fit deconvolution spectrum (Supplementary Figure S1). Figure 2B corresponds to a selected TEM micrograph, demonstrating the presence of spherical silver nanoparticles in PVA hydrogels. Histograms of the nanoparticle size distribution are shown in Figure 2C. The superposed red dotted line represents the Gaussian fit, from which an average silver nanoparticle size of 14.5 ± 2.5 nm is obtained.

### 3.2. PVA and PVA-AgNPs Hydrogel’s Microstructure

**Figure 3:**
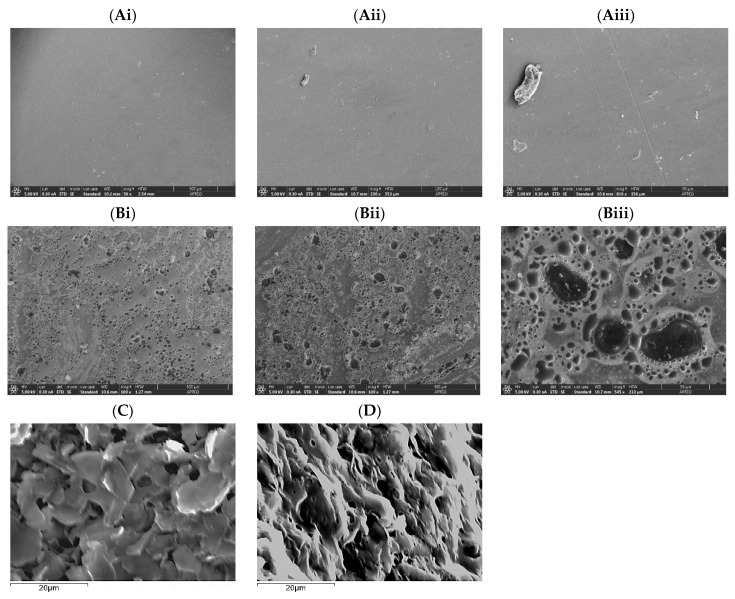
Representative SEM micrographs of the hydrogels PVA (**A**) and PVA-AgNPs (**B**); ((**i**–**iii**) represent different magnifications at 500 µm, 100 µm, and 50 µm, respectively) freeze-dried hydrogels of PVA (**C**) and PVA-AgNPs (**D**); Scale bar: 20 µm.

The microstructure of the PVA and PVA-AgNPs hydrogels is presented in Figure 3. The control PVA hydrogels had smooth and even surfaces; in contrast, more porous and uneven surfaces were found in AgNPs-loaded PVA hydrogels (Figure 3A,B). We also investigated the microstructures of freeze-dried hydrogels, and the images showed more interconnected porous structures in both hydrogels (Figure 3C,D). In addition, the SEM images demonstrated a porous microstructure, porosity, and pore shape of the samples. Micropores, ranging from 1 mm to 10 mm, were observed for the PVA-AgNPs hydrogel. On the other hand, the PVA-hydrogel showed a larger pore size with a narrow distribution of around 5–10 mm, as compared to the PVA-AgNPs hydrogel.

### 3.3. Water-Holding Capacity

**Figure 4:**
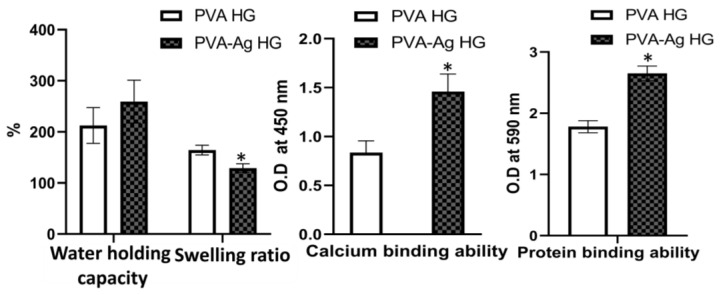
Functional properties of silver nanoparticle-loaded PVA hydrogels. PVA HG: Poly (vinyl alcohol) hydrogel; PVA-Ag HG: Poly (vinyl alcohol) hydrogel loaded with silver nanoparticles. * *p* < 0.05 vs. PVA HG.

The ability of the hydrogel to hold water in intramolecular space was investigated using the weight difference before and after immersion in water, as seen in Figure 4. The results showed that both hydrogels had an excellent water retention capacity, and the PVA hydrogel showed a higher water retention ability than AgNPs-PVA-HG; however, the significance was not statistically significant. The water-holding capacity of hydrogels was 212.48 + 35 and 259.21 + 42% for the PVA hydrogel and AgNPs-PVA-HG, respectively.

### 3.4. Swelling Ratio

Though the water-holding capacity of both hydrogels did not change significantly, the pattern of swelling ratio differed between the PVA hydrogel and AgNPs-PVA-HG. As shown in Figure 4, the swelling ratio of the hydrogel decreased in AgNPs-PVA-HG when compared to the PVA hydrogels. The swelling ratio of the PVA hydrogel and AgNPs-PVA-HG was 164.37 ± 16.45 and 129.18 ± 21.28%, respectively.

### 3.5. Calcium-Binding Ability

The effect of hydrogels in support of extracellular matrix formation was investigated by treating the hydrogels with calcium, and the ability of the hydrogels to bind with minerals was investigated by the alizarin red-S staining method.

**Figure 5:**
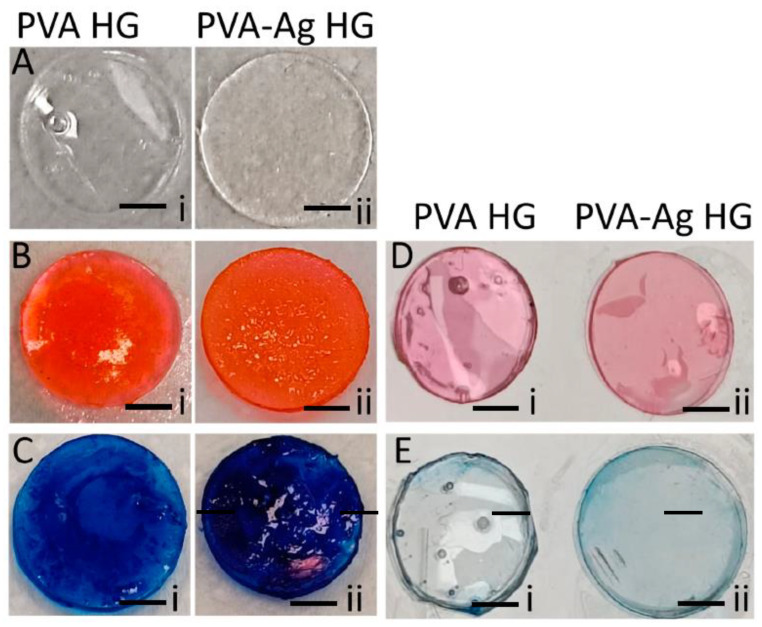
Calcium-binding and protein-binding ability of silver nanoparticle-loaded PVA hydrogels. i—PVA HG: Poly (vinyl alcohol) hydrogel; ii—PVA-Ag HG: Poly (vinyl alcohol) hydrogel loaded with silver nanoparticles. (**A**) simple hydrogels without any treatment; (**B**) hydrogels with alizarin red-S staining after calcium treatment; (**C**) hydrogels with CBB-G250 staining after FBS treatment; (**D**,**E**) hydrogels after alizarin red-S staining and CBB-G250 staining without calcium loading, respectively. Scale bar: 0.3 cm.

Surprisingly, the rate of mineral deposition significantly increased when adding AgNPs to the hydrogels (*p* < 0.05) (Figure 5B). The rate of calcium binding of AgNPs-PVA-HG was 1.46, and the PVA hydrogel had a calcium-binding rate of 0.836 (Figure 4). In order to investigate the background staining of the hydrogels with alizarin red-S staining, the hydrogels without calcium treatment (in parallel) were also stained with alizarin red-S stain. The results showed that the hydrogels with or without AgNPs did not show any significant positive stain with alizarin red-S (Figure 5D).

### 3.6. Protein-Binding Ability

In order to investigate the potential compatible interaction of a hydrogel with cellular protein receptors, we have estimated the ability of the hydrogels in their protein-binding ability. To do this, the hydrogels were incubated with fetal bovine serum, and the hydrogel-bounded protein was quantified using the CBB G-250 method (Figure 5C,E). Similar to the calcium-binding ability of hydrogels, the protein-binding rate of the hydrogels was also improved upon by incorporating AgNPs (Figure 5C). The hydrogel with AgNPs potentially improved regarding protein adhesion, which was proved by the higher staining rate of CBB G-250 after FBS treatment when compared to the PVA hydrogel. The protein-binding rate of PVA hydrogel (without AgNPs) and AgNPs-PVA-HG was 1.78 and 2.65, respectively (Figure 4). On the other side, the hydrogels without FBS treatment were also stained with CBB G-250 in order to investigate the background staining of the hydrogels. The results showed that the hydrogels with or without AgNPs did not show any significant positive stain with CBB G-250 (Figure 5E).

### 3.7. Cell Proliferation

As the hydrogels showed excellent functional properties, the biocompatibility using MSCs was investigated for 1, 3, and 7 days of treatment with hydrogels. In general, cell proliferation increased as expected with culture time, i.e., the cells cultured for 7 days had a higher proliferation rate compared to the day 1 and day 3 cultures (*p* < 0.05). On day 1, a similar proliferation rate for the MSCs was observed between PVA hydrogel and the control (without hydrogel); on the other hand, the proliferation rate of MSCs improved in AgNPs-PVA-HG. On day 3, the cells cultured on the PVA hydrogel (*p* < 0.05) and AgNPs-PVA-HG (*p* < 0.01) had more proliferative effects than the control cells. Among the hydrogels, AgNPs-PVA-HG had a higher proliferation rate regarding MSCs on day 3 and day 7.

### 3.8. Cytotoxicity

**Figure 6:**
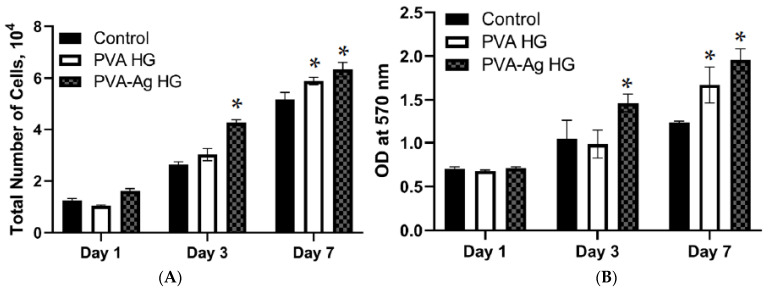
Cell proliferation (**A**) and cytotoxicity (**B**) of silver nanoparticle-loaded PVA hydrogels. PVA HG: Poly (vinyl alcohol) hydrogel; PVA-Ag HG: Poly (vinyl alcohol) hydrogel loaded with silver nanoparticles and control cells cultured without hydrogels. * *p* < 0.05 vs. control.

Similar to proliferation, cell viability increased in relation to culture time in all the groups, and higher cell viability was observed on day 7 than on day 1 (Figure 6). There was no significant difference in cell viability between the control and hydrogel-cultured cells on days 1 and 3. However, cell viability was higher in the hydrogel-cultured cells when compared to the control on day 7. Similarly, among the hydrogels, no significant changes in cell viability were observed on days 1 and 7; in contrast, the cells cultured on AgNPs-PVA-HG showed more viability than PVA hydrogel on day 3.

### 3.9. Histological Staining

**Figure 7:**
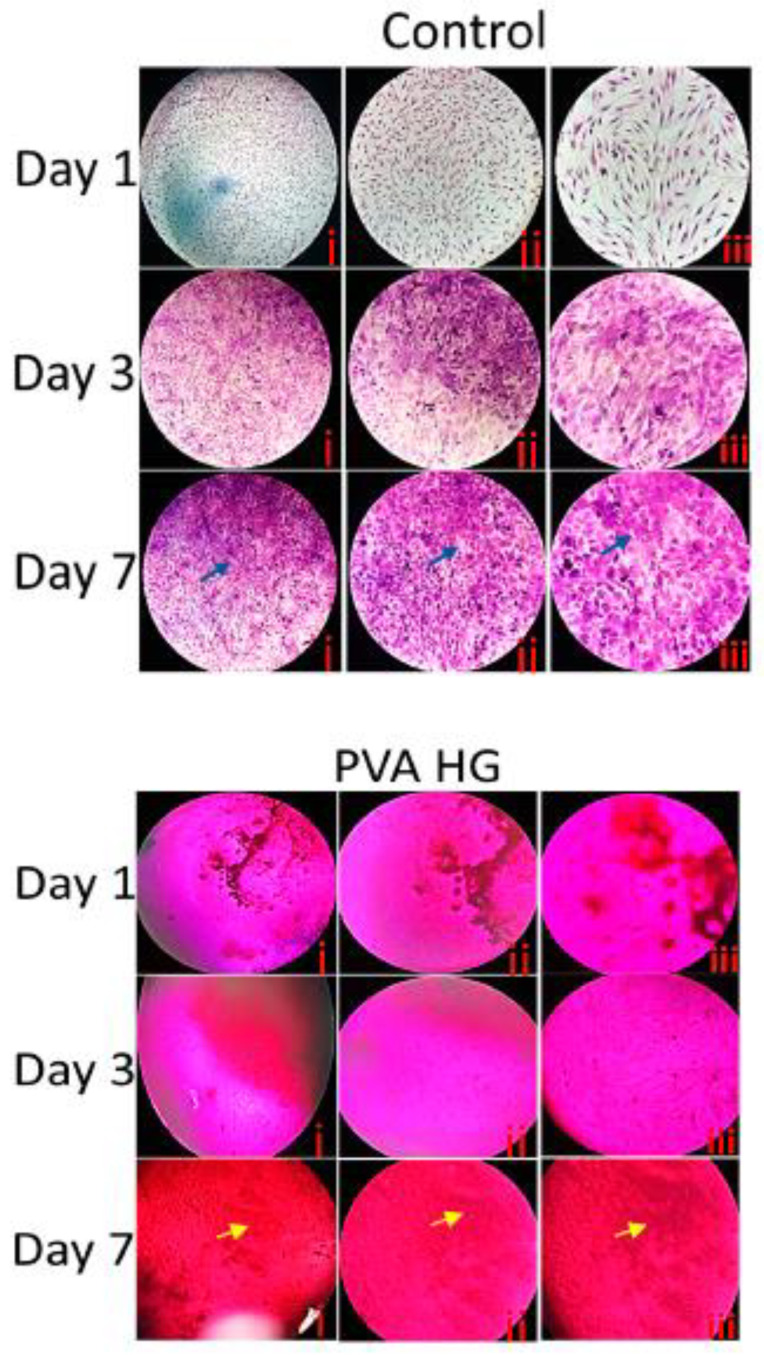

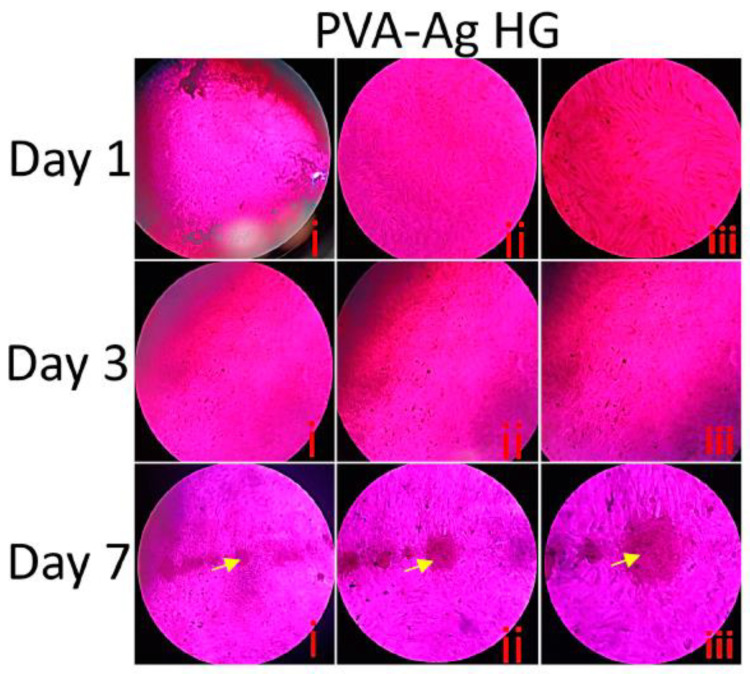
Histological staining of MSCs cultured on silver nanoparticle-loaded PVA hydrogels. PVA HG: Poly (vinyl alcohol) hydrogel; PVA-Ag HG: Poly (vinyl alcohol) hydrogel loaded with silver nanoparticles and control cells cultured without hydrogels. i, ii, and iii correspond to the different magnifications at 4×, 10×, and 20×, respectively. The scale bar corresponds to i—200 µm, ii—100 µm, and iii—50 µm. Blue and Yellow arrows show the densely populated cells and extracellular matrix deposition, respectively.

**Figure 8:**
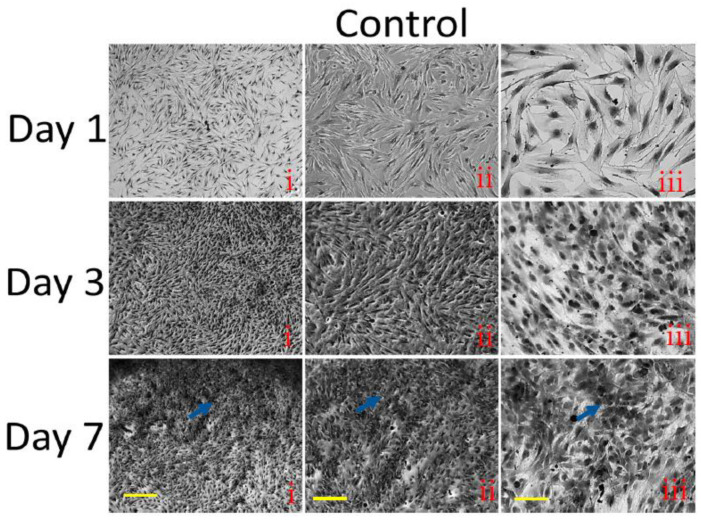

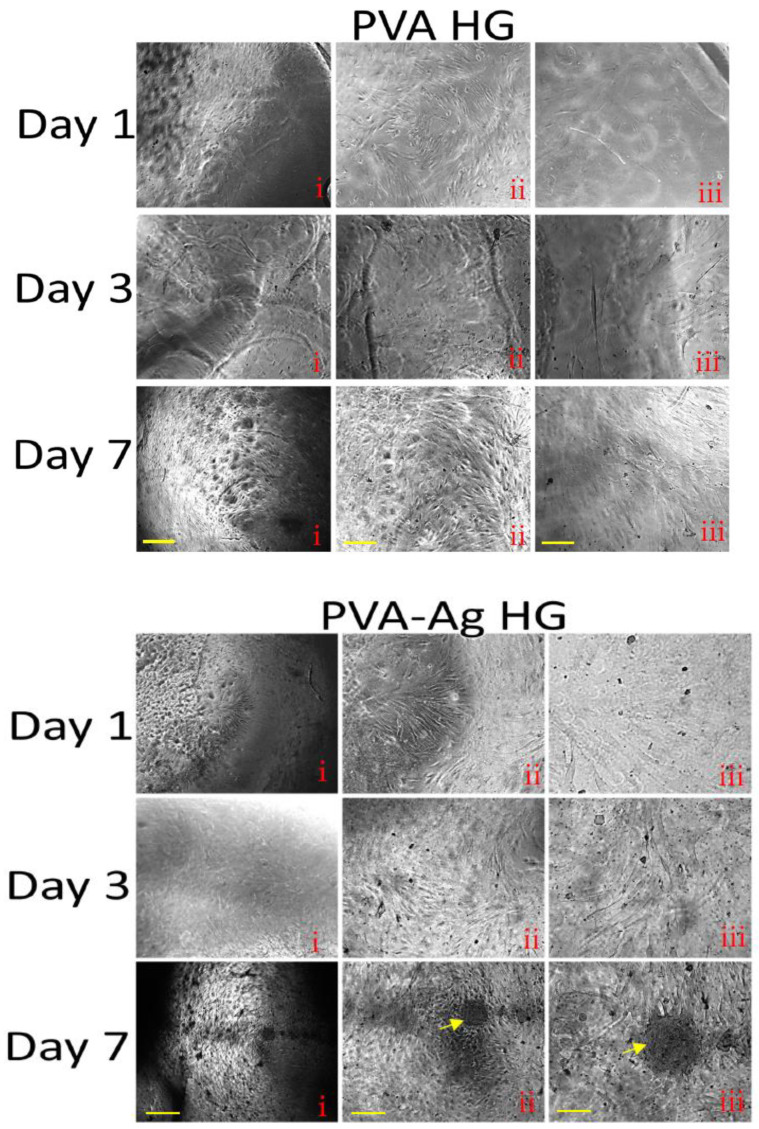
Light microscopic pictures of MSCs cultured on silver nanoparticle-loaded PVA hydrogels. PVA HG: Poly (vinyl alcohol) hydrogel; PVA-Ag HG: Poly (vinyl alcohol) hydrogel loaded with silver nanoparticles and control cells cultured without hydrogels. From left to right, different magnifications at i—4×, ii—10×, and iii—20× are shown. The scale bar corresponds to i—200 µm, ii—100 µm, and iii—50 µm. Blue and Yellow arrows show the densely populated cells and extracellular matrix deposition, respectively.

The effect of hydrogels on MSC proliferation and cytotoxicity was further investigated by light and histological (H&E staining) microscopy. As shown in Figure 7 and Figure 8, the cells were more populated as culture time increased from day 1 to day 7.

In the light microscopy, the cells stained with H&E stains clearly showed the presence of a cytoskeleton and the fibrillar structures of the cellular components. On day 1, the cells cultured without hydrogels were more fibrillar bipolar structures compared to day 3 and day 7. In contrast, the cells cultured without hydrogels for 3 and 7 days showed a more dense fibrillar structure, which was more pronounced on day 7. When compared to the control cells, the cells cultured on hydrogels showed different morphology and more cell clumps were observed on hydrogel-cultured cells. Among the hydrogel groups, the H&E staining showed more fibrillar cells in AgNPs-PVA-HG. Interestingly, the cells cultured on AgNPs-PVA-HG for 7 days potentially produced extracellular matrix deposition (yellow arrows) and more cell clumps compared to the PVA hydrogel. In addition, the light microscopic pictures clearly showed that the cells were firmly adherent to the hydrogels, and the cell’s morphology was different between the control (without hydrogels) and hydrogels groups, showing more fibrillar structures even on day 3 and day 7 in hydrogel groups; in contrast, the cells become more aggregated with less fibrilar structures for those cultured in the control group. The extracellular matrix formation of MSCs cultured on AgNPs-PVA-HG was clearly seen in the light microscopic picture.

### 3.10. Fluorescence Microscopy

**Figure 9:**
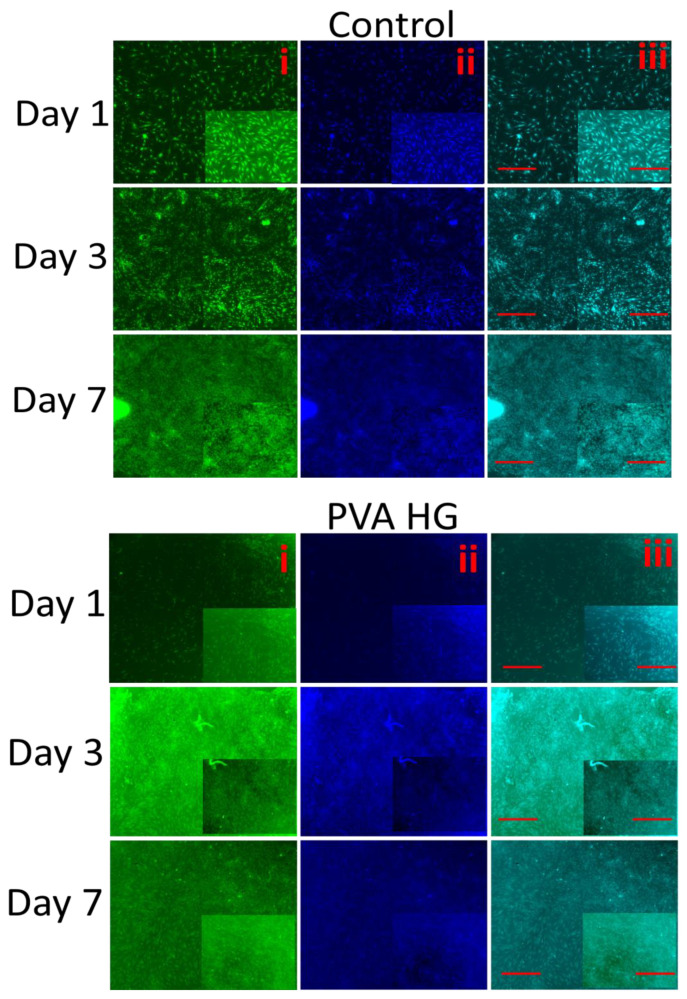

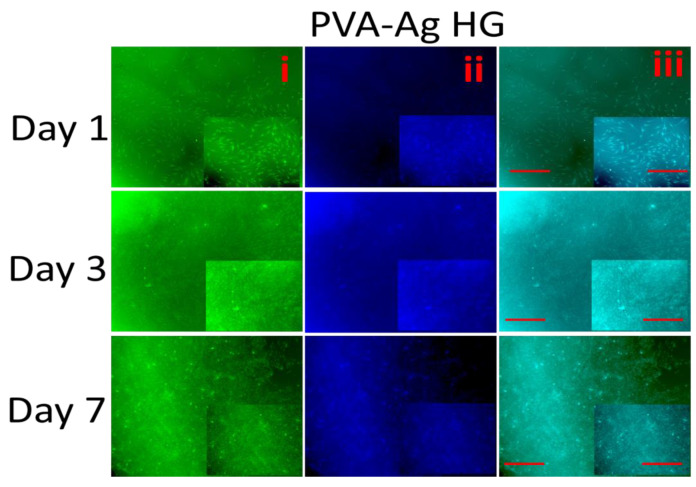
Fluorescence microscopic pictures of MSCs cultured on silver nanoparticle-loaded PVA hydrogels. PVA HG: Poly (vinyl alcohol) hydrogel; PVA-Ag HG: Poly (vinyl alcohol) hydrogel loaded with silver nanoparticles and control cells cultured without hydrogels. i—cells stained with FITC, ii—cells stained with DAPI, and iii—merged image. Images were captured at 4× and 10× magnification (insert). Scale bar: 200 µm and 100 µm (insert).

We further evaluated the proliferative and biocompatibility effect of hydrogels on MSCs through FITC and DAPI fluorescence microscopy. As observed in H&E staining and light microscopy, the fluorescent intensity of FITC and DAPI significantly increased in the control cells cultured for 7 days when compared to the cells cultured for 1 and 3 days (Figure 9). Similar to the control, the cells cultured on hydrogels for 7 days had high FITC and DAPI staining intensity compared to the day 3 and day 1 cultures. Among the hydrogel-cultured cells, the DAPI staining (nucleus) of MSCs was higher in cells cultured on AgNPs-PVA-HG compared to cells cultured on PVA hydrogels for 7 days. Interestingly, in each period, the level of fluorescence staining was highly upregulated in the cells cultured on hydrogels compared to the control cells (without hydrogels), and the upregulating effect was highly pronounced in cells cultured on AgNPs-PVA-HG.

## 4. Discussion

In the present study, the content of PVA- and glycerine-modulated mechanical properties, such as elasticity, yield strength, young modulus, and concomitant physicochemical properties, were analyzed. Indeed, more glycerine will provide more flexibility and serve to modulate the intrinsic micro and macroporosity structure, which are of paramount importance in tissue engineering applications [29]. The maximum absorbance of AgNPs was observed at 409 nm, with a peak at half maximum (PWHM) of 81 nm, which is typical for spherical AgNPs [24,25]. The average silver nanoparticle size (AgNP) of 14.5 ± 2.5 nm was obtained from TEM observation, which was in total agreement with the previous literature [24,25]. The optical properties of AgNPs were previously studied by Amirjani et al. [30]. In another study, a hydrogel fabricated using an AgNP-loaded porcine dermal extracellular matrix (PADM) exhibited a single peak at ~400 nm, which is similar to the present study. The authors claimed that the AgNPs were uniformly distributed in the PADM hydrogels and were not agglomerated [31]. As shown in Supplementary Video S1, the fabricated hydrogels were very flexible and strong, which makes them more suitable for biomedical applications.

Previously, Wang et al. tested the water-holding capacity of cellulose-based hydrogel copolymerized with polyacrylic acid (PAA)-grafted quaternized cellulose (QCE) and PVA at different pH (pH 3, 5, 7, 9, and 11), and also different QCE and PVA contents from 0% to 1.5%, and 0% to 8%, respectively. The authors concluded that the water adsorption behavior of the hydrogel was significantly affected by the pH range and the abundant carboxyl groups of PAA. The authors also stated that the water adsorption capacity of the hydrogel decreased as the PVA content increased [32]. Similarly, Tanpichai and Oksman reported that the water-holding capacity of a hydrogel was reduced from 97.1 to 81% as the PVA concentration increased from 2 to 12%, respectively. The authors further claimed that the hydrogel’s water content depends on the solid content in the hydrogels i.e., the water content in the hydrogels decreases with greater solid content [33].

Similar to the present study, the swelling ratio of cellulose nanocrystal (CNC)-reinforced PVA was tested by Tanpichai and Oksman, and they found that the swelling ratio of the PVA (4%) hydrogel was 577%, which was higher than the present study. The authors further reported that the swelling ratio of the hydrogel was reduced from 577 to 165% as the PVA concentration increased from 4 to 12%, respectively [33]. Several reports explained a possible hypothesis for this effect. For instance, the hydrophilic ability of the functional groups regulates the swelling ratio of the hydrogel [34]. The hydrogel swelling ratio increased due to decreasing entanglement and the cross-linking density of polymer chains [35]. In order to support this evidence, the availability of the functional groups in the hydrogels, which interact with water, decreased the cross-linking rate of the PVA matrix and the cellulose, leading to the lower swelling degree of the hydrogels [34,36].

It has been reported that the swelling behavior of synthesized nanocomposites was significantly altered by the presence of nanosilver in the polymer network [37]. Similar to the present study, several reports claimed the diminishing effect of AgNPs in the swelling properties of hydrogels. The major reason is the cross-linking of the hydroxyl group (−OH) of PVA with silver ions, thereby reducing the interaction between PVA and water molecules. The other reason we thought of is the silver nanoparticle in the gel would occupy the mesh space or pore volume of the gel network, resulting in a decrease in the swelling ratio of the gel. These reasons diminish the rate at which water diffuses or penetrates the gel. Similar results were observed from the previous study in the ex situ polymerization of APECAg gel [38].

The ability of the mineral-binding rate of hydrogel was potentially improved by AgNPs, which ultimately shows the efficiency of the hydrogels in extracellular matrix deposition during bone formation. For instance, PVA hydrogels were fabricated with biomimetic mineral hydroxyapatite or biomimetic apatite for cartilage repair [39,40]. In order to support this evidence, several approaches have been initiated to investigate the bone and cartilage regeneration ability of PVA hydrogels combined with pectin [41]; polyacrylamide, bioactive glass, and halloysite nanotubes [42]; alginate/biphasic calcium phosphate [43]; hydroxyethyl chitosan/biphasic calcium phosphate [44]; gelatin [45,46]; chitosan [47], and graphene oxide [48].

In addition, the calcium biomineralization of a PVA hydrogel was sufficiently proved by several studies [42,43,47]. We speculate that the mineral deposition on a PVA hydrogel could be due to the possible interaction between the hydroxyl functional groups of PVA and calcium. In order to support this hypothesis, the interaction between hydroxyl and Ca-rich hydroxyapatite was studied by Wang et al. using a computational method [49]. It has been reported that the hydroxyl group possibly binds with Ca-rich hydroxyapatite through electrostatic interaction [50,51]. In the present study, both PVA hydrogel and AgNPs-PVA-HG showed excellent mineral-binding ability, which proves the applicability of this hydrogel in bone and cartilage regeneration.

The microstructural properties revealed that the presence of these micropores could be attributed to the interactions between AgNPs and the rapid sublimation process occurring during freeze-drying, promoting reduced pressure surrounding the nanoparticles. It is worth noting that, at this scale, EDX was unable to provide a microanalysis to verify the presence of the silver nanoparticles, which demonstrated that AgNPs are homogeneously distributed through the 3D structure as an individual or in a small cluster of nanoparticles. The microstructure (porosity, shape, interconnected structure, etc.) of hydrogel had a great influence on tissue engineering applications because it allows for cell intrusion, adhesion, and proliferation. Additionally, high interconnectivity promotes uniform cell distributions as well as the diffusion of nutrients to and from the cell substrates. Thus, in the PVA-AgNPs hydrogel, the existence of a distribution of micropores would be favorable in terms of biocompatibility since it increased the interconnectivity and interface area of the porous structure.

The applicability of PVA hydrogels in biological cell cultures (PVA-cell receptor interaction) was indirectly determined by the absorption of plasma protein by PVA hydrogel [15,52,53]. Hence, the PVA hydrogels were treated with FBS, and the level of protein binding was measured by CBB G-250. Interestingly, both hydrogels favored the adsorption of protein, and the protein-binding ability was accelerated by AgNPs. Similar to the present study, the protein adsorption of PVA hydrogel was reported to be fabricated with hydroxyapatite-chitosan [54], alginate/dextran [55], 1-Vinyl-3-butylimidazolium bromide and acrylamide [56,57], and retinol [15]. In the present study, the protein adsorption of PVA hydrogel depends on the surface energy, surface hydrophobicity chemical groups, and electrostatic interaction between the proteins and the hydrogel surface [58,59,60]. In order to support this evidence, an earlier study claimed that the surface protein absorption of hyaluronic acid-based nanofibers were loaded onto PVA hydrogel via physical bonds, such as hydrophobic interactions, ionic bonding, hydrogen bonding and Van der Waal interactions [56].

In addition to the PVA hydrogel potential in protein adsorption, the accelerated rate of protein adsorption of AgNPs could be explained by several reports. The interaction of nanoparticles with protein in the biological environment produces a nanoparticle–protein corona complex, which is the primary cause of in vivo biodistribution and pharmacokinetic profiles [61,62]. The cellular uptake of NPs mainly depends on the interaction between the NPs and cellular proteins [63,64,65]. Baimanov et al. reviewed the possible interactions of nanoparticle–protein on the biological behavior and applications of therapeutic nanomaterials [66]. Previously, Monteiro-Riviere et al. investigated the effect of pre-exposure incubation with three different proteins (human serum albumin, IgG, and transferrin) on the human epidermal keratinocyte uptake of different AgNPs [67]. In order to support the above study, Kettler et al. also reported that the ability of AgNP uptake by monocytic THP-1 cells depends on the presence of serum proteins and particle size [68]. All these studies revealed that AgNPs could potentially support the protein adsorption of biomaterials in both in vitro and in vivo models.

In vitro studies, such as proliferation, cytotoxicity, histological staining, and fluorescence staining, showed that the growth of the cell was superiorly supported through culturing the cells on hydrogels than the control, and the effect was more pronounced in cells cultured on AgNPs-PVA HG. Similar to the present study, earlier studies proved the proliferative effect of PVA hydrogel in MSC cell growth [15,69,70,71]. In addition, the proliferative effect of MSCs by AgNPs-PVA-HG was also reported by several researchers [72,73,74]. Similar to the present study, the in vitro responses of mesenchymal stem cells, the in vivo immunomodulatory and healing behavior of a PVA membrane containing AgNPs, and collagen and hyaluronic acid were reported by Júnior et al. [72]

The hydrogel fabricated with AgNPs and the chitosan-PEG mixture showed excellent healing properties for treating diabetic chronic wounds [75,76]. Additionally, the antibacterial and wound-healing ability of PVA/cellulose/AgNP hydrogels were studied by Song et al. [77], and they reported that the proliferative effect of L929 cells was upregulated more by PVA/cellulose/AgNP silver hydrogels than the control, and concluded that the incorporation of AgNPs into PVA/cellulose could increase the formation of three-dimensional networks [78,79]. Based on the above findings, we opined that AgNPs-PVA-HGs promote the proliferation of MSCs by liberating the AgNPs from PVA hydrogels, which potentially enter into cells through two fundamental biological processes, such as phagocytosis and pinocytosis [68,80,81].

The actual signaling mechanism of AgNPs in cell proliferation was reported by several authors. For instance, AgNPs potentially alter the gene expression that is involved in multiple cellular pathways, including cell proliferation and hormone signaling pathways, such as tumor necrosis factor (TNF), matrix metalloproteinases, and interleukin (IL)-12 and IL-1 [82].

**Figure 10:**
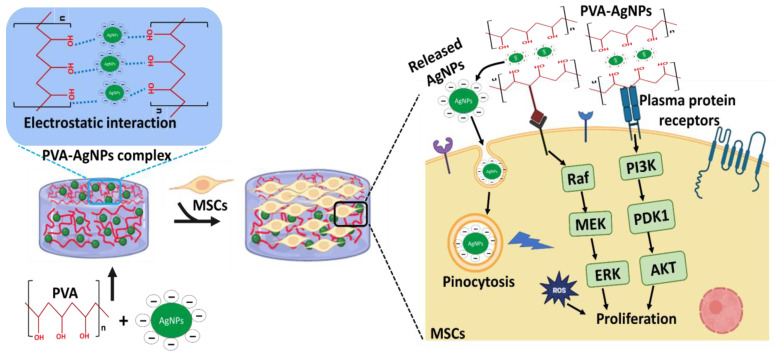
Complex interactions of PVA and AgNPs in hydrogel and molecular signaling mechanism of AgNP-loaded PVA hydrogels in MSC proliferation.

More detailed molecular signaling mechanisms of AgNPs in cell proliferation were reported by Li et al. [83] by treating human skin fibroblasts with AgNP-doped chitosan oligosaccharide/PVA nanofiber. The authors evidenced that AgNPs upregulated the proliferation of skin fibroblasts by (1) stimulating cell cycle progression from G1 into the S and G2 phases, (2) reducing the proportion of cells in the G0/G1 phase, inducing S and G2/M arrest, and (3) upregulating the cell factors associated with the TGF-β1/Smad signal transduction pathway, such as TGF-β1, TGFβRI, TGFβRII, pSmad2, pSmad3, collagen I, collagen III, and fibronectin. In another study, the induction of neural differentiation of SH-SY5Y cells by biologically synthesized AgNPs was investigated, and it was concluded that biologically synthesized AgNPs altered the cell morphological changes and neurite length of SH-SY5Y cells by triggering neuronal differentiation marker expression, such as Drd-2, Gap-43,neurogenin-1, synaptophysin, β-tubulin III, and Map-2, followed by upregulating the intracellular reactive oxygen species (ROS), the activation of several kinases, such as ERK and AKT, and downregulating dual-specificity phosphatase (DUSP) expression in AgNP-treated SH-SY5Y cells [84]. Collectively, AgNPs-PVA-HG could support stem cell proliferation by modulating several cellular signaling pathways, like ERK, AKT, Map-2, and TGF-β1/Smad signals in biological cells (Figure 10).

## 5. Conclusions

In the present study, the functional properties and in vitro biological response of AgNPs-PVA-HG were investigated. Our study clearly demonstrated that the functional properties, such as protein adsorption and the calcium-binding abilities of PVA hydrogel, increased due to AgNPs, and the swelling properties of PVA hydrogel decreased due to AgNPs incorporation. The prominent protein and mineral affinity of AgNPs directly proved the efficacy of the fabricated composite PVA hydrogel in regenerative tissue applications. Further, the biocompatibility of the composite hydrogels in culturing MSCs was investigated. In general, the proliferative rate of MSCs increased with AgNPs-PVA-HG treatment. Histological and fluorescence images proved that the MSCs adhered to both the PVA hydrogel and AgNPs-PVA-HG more efficiently, and the cells cultured for 7 days on AgNPs-PVA-HG deposited extracellular matrix. Based on the above findings, this study concludes that the fabricated AgNPs-PVA-HG could be an effective system for culturing mammalian cells, especially for stem cell differentiation. However, further studies are essential to prove the biological role of this system in regenerative tissue applications.

## Data Availability

Not applicable.

## Supplementary Materials

The following supporting information can be downloaded at: https://www.mdpi.com/article/10.3390/pharmaceutics15071843/s1, Figure S1. Peakfit deconvolution spectrum synthesized silver nanoparticles. Video S1. The flexible and elastic properties of fabricated hydrogels.
