# Supplementary material for: Protein Adsorption, Calcium-Binding Ability, and Biocompatibility of Silver Nanoparticle-Loaded Polyvinyl Alcohol (PVA) Hydrogels Using Bone Marrow-Derived Mesenchymal Stem Cells
Source: Pharmaceutics. 2023 Jun 28;15(7):1843. doi: 10.3390/pharmaceutics15071843 (PMC10384843; doi:10.3390/pharmaceutics15071843)
Supplement: Supplementary file 1 [file pharmaceutics-15-01843-s001.zip › pharmaceutics-2441137-supplementary.pdf]

Supplementary Materials: Protein adsorption, Calcium-binding Abilities and Biocompatibility of Silver Nanoparticles Loaded Polyvinyl alcohol (PVA) Hydrogels Using Bone Marrow-derived Mesenchymal Stem Cells

Jeevithan Elango, Camilo Zamora-Ledezma, Frank Alexis, Wenhui Wu, José Eduardo Maté Sánchez De Val

Supplementary information:

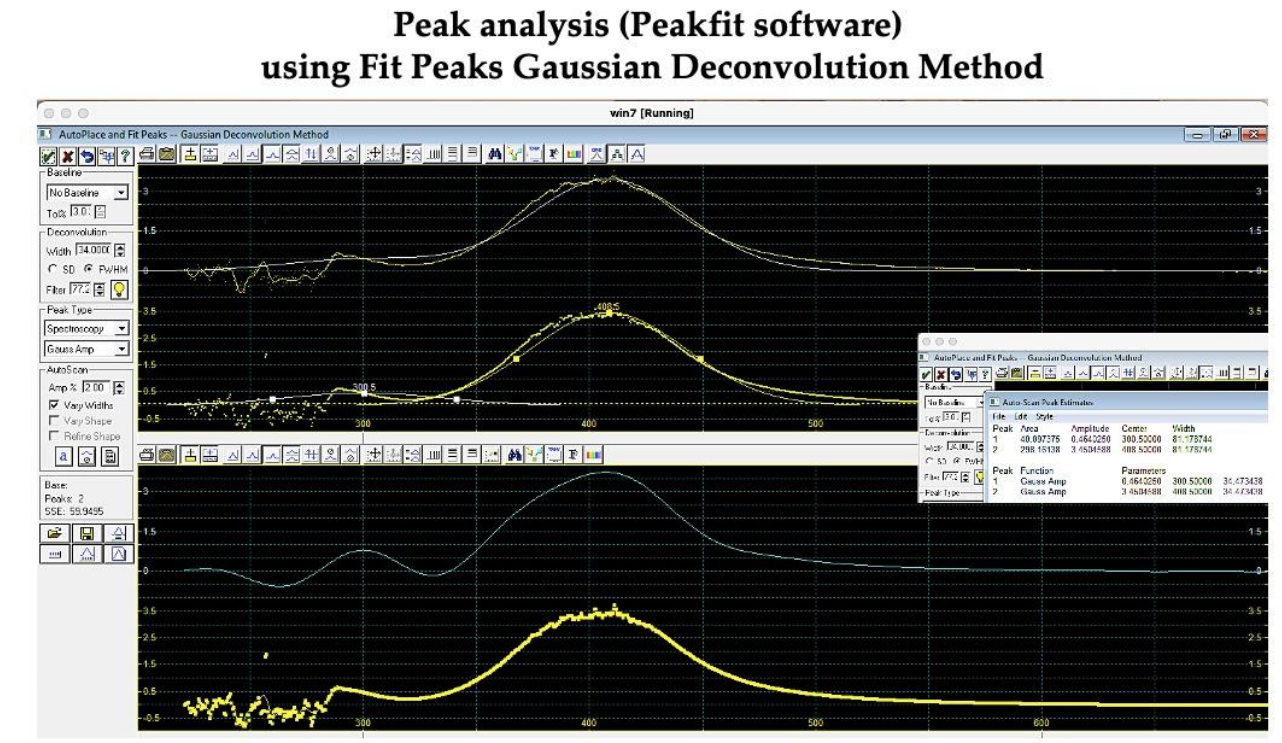

Figure S1. Peakfit deconvolution spectrum synthesized silver Nanoparticles.
